# Evaluation of maxillary canine and molar movement during the first phase of extraction space closure: a multilevel analysis

**DOI:** 10.1590/2177-6709.28.4.e232338.oar

**Published:** 2023-09-15

**Authors:** Luiz Gonzaga GANDINI, Patricia Pigato SCHNEIDER, Ki Beom KIM, André da Costa MONINI, Helder Baldi JACOB

**Affiliations:** 1 Universidade Estadual Paulista (UNESP), School of Dentistry, Department of Orthodontics (Araraquara/SP, Brazil).; 2 Saint Louis University, Center for Advanced Dental Education, Department of Orthodontics (Saint Louis/Missouri, USA).; 3 Private practice (Goiânia/GO, Brazil).; 4 The University of Texas, School of Dentistry, Department of Orthodontics, Health Science Center (Houston/Texas, USA).

**Keywords:** Orthodontic space closure, Orthodontic tooth movement, Orthodontic anchorage procedure, Canine tooth

## Abstract

**Objective:**

This study was designed to analyze and compare the cusp and apex movements of the maxillary canines and first molars during canine retraction in the first step of extraction space closure, and to evaluate if these teeth follow a curvilinear (acceleration and/or deceleration) movement rate.

**Material and Methods:**

Twenty-five patients (23.3 ± 5.1 years of age) were enrolled. The retraction of the maxillary canines was accomplished using nickel-titanium closed coil springs (100gf) on 0.020-in stainless steel archwire. Oblique cephalograms were traced and superimposed on the anatomic best fit of the maxilla (before the retraction [T0], and after one month [T1], three [T3], five [T5] and seven [T7] months). Statistics was based in a normally distributed data. Multilevel procedures were used to derive polynomials for each of the measurements. Student’s t-test and one-way repeated measures ANOVA were conducted. The level of significance of 5% was adopted.

**Results:**

Canine cusps and apexes did not follow a quadratic curve regarding horizontal movement (neither accelerate nor decelerate). Canine and molar cusps showed more horizontal movement than apexes (4.80 mm vs. 2.78 mm, and 2.64 mm vs. 2.17 mm, respectively).

**Conclusions:**

Canine did not accelerate or decelerate overtime horizontally; the cusps and apexes of the canines and molars showed more horizontal movement and larger rate at the beginning of canine retraction, followed by significantly smaller and constant movement rate after the first month.

## RESUMO

**Objetivo:**

O objetivo deste estudo foi analisar a movimentação das cúspides e dos ápices de caninos e primeiros molares superiores durante a retração de caninos na primeira fase do fechamento do espaço pós-extração, e avaliar se esses dentes apresentam mudança na taxa de movimentação (aceleração e/ou desaceleração).

**Material e Métodos:**

Vinte e cinco pacientes (idade = 23,3 ± 5,1 anos) foram incluídos. A retração dos caninos superiores foi realizada com molas helicoidais fechadas de níquel-titânio (100gf) em arco 0,020” de aço inoxidável. Foram feitos traçados cefalométricos de telerradiografias oblíquas e sobrepostos ao melhor ajuste anatômico da maxila (antes da retração [T0]; após um mês [T1], três meses [T3], cinco meses [T5] e sete meses [T7]). A estatística foi baseada em dados com distribuição normal. Procedimentos multiníveis foram usados para derivar polinômios para cada uma das medidas. Teste *t* de Student e ANOVA de uma via para medidas repetidas foram realizados, adotando-se um nível de significância de 5%.

**Resultados:**

As cúspides e os ápices dos caninos não seguiram uma curva quadrática em relação ao movimento horizontal (sem aceleração ou desaceleração). As cúspides dos caninos e molares apresentaram mais movimento horizontal do que os ápices (4,80 mm vs. 2,78 mm e 2,64 mm vs. 2,17mm, respectivamente).

**Conclusões:**

A movimentação horizontal dos caninos não acelerou ou desacelerou ao longo do tempo; as cúspides e os ápices dos caninos e molares apresentaram maior movimento horizontal e maior taxa de movimentação no início da retração dos caninos, seguida por uma taxa de movimentação significativamente menor e constante após o primeiro mês.

## INTRODUCTION

The space closure is required during the orthodontic treatment after premolar extraction therapy.^1^ Traditionally, the two-step retraction (TSR) is the biomechanical strategy used to close the spaces in which the canines are individually retracted, followed by the incisors.^2^ This technique is believed to preserve posterior anchorage.^3^ However, it has been associated with longer treatment time.^4^

In relation to individual canine retraction, the orthodontic literature reports high variability of the monthly movement rate of this tooth, ranging from 0.71 to 1.85 mm under sliding mechanics (Table 1).^5^ ^-^ ^12^ This variation can be due to study design, since dental casts do not allow the measurement of the apical tooth movements, and lateral radiographs overlap the patient’s right and left sides on the same image. To overcome this drawback, the 45° projection lateral cephalometric radiograph has been suggested to evaluate the treatment responses, because it allows tooth visualization without superimposing the opposite side.^13^ Nonetheless, currently, only one study investigated the canine movements using this method,^12^ and the anchorage loss rates during its retraction has been reported in only one study until now.^8^

**Table 1:** Human studies on maxillary canine and molar movement rates with sliding mechanics.

Author	Sample size / Age range	Force sources	Force magnitude	Canines movement rate (mm/mo)	Molars movement rate (mm/month)	Method
Dixon et al.^7^	n = 33 / 10 to 18 years	Active ligatures	-	0.35	N/A	Models
		Powerchain	-	0.58		
		NiTi closed-coil spring	200gf	0.81		
Nightingale and Jones^5^	n = 8 / 12 to 18 years	Elastomeric chain	209-109gf	0.84	N/A	Models
		NiTi closed-coil spring	300-149gf	1.04		
Hayashi et al.^6^	n = 8 / 19.4 to 29.2 years	NiTi closed-coil spring	100gf	1.41	N/A	Models
		Ricketts spring	100gf	1.91		
Bokas and Woods^8^	n = 12 / 13 to 14.5 years	NiTi closed-coil spring	200gf	1.85	0.46	Models
		Power chain	-	1.68	0.45	
Herman et al.^9^	n = 16 / 11.4 to 22.6 years	NiTi closed-coil spring	150gf	1.3 (MA)	N/A	Models
Thiruvenkatachari et al.^10^	n = 10 / 16 to 20 years	NiTi closed-coil spring	100gf	0.93 (MA)	N/A	Lateral Cephalogram
				0.81 (CA)		
Oz et al.^11^	n = 19 / 12.7 to 15.3 years	NiTi closed-coil spring	200gf	0.91 (SLB)	N/A	Lateral Cephalogram
				0.85 (MT)		
Monini et al.^12^	n = 25 / 17 to 35 years	NiTi closed-coil spring	100gf	0.71 (SLB)	N/A	Cephalogram at 45º
				0.72 (CB)		

N/A = information not available; MA = mini-implant anchorage; CA = conventional anchorage; SLB = self-ligation bracket; MT = Modified Twin Bracket; CB = conventional bracket.

Despite the clinical relevance of this subject, the tooth movement rates reported in the aforementioned studies^5^ ^-^ ^12^ were calculated based on the changes that occur just between two observation-periods. Only by means of multiple records it is possible to create a movement curve by multilevel estimates that would be able to show the real tooth movement rate velocity with precision during the follow-up time of retraction phase.^14^ ^-^ ^16^ Therefore, tooth movement rates should be evaluated by means of records obtained at several times of treatment, and curve fitting procedures should be used to determine the real tooth movement rate.^14^ ^-^ ^16^ Polynomial is a multiple linear equation that quantifies the form of the growth curve, making no assumptions about the shape of the actual curve. A linear polynomial is a straight line (tooth movement rate velocity) and a quadratic polynomial describes a curve (tooth movement rate velocity with acceleration or deceleration).^14^ ^,^ ^15^ Multilevel estimates have been shown to be more stable and meaningful than estimates based on ordinary least squares.^16^

The present study was designed to analyze the cusp and apex movements of the maxillary canines and first molars during canine retraction in the first phase of extraction space closure associated with the two-step retraction (TSR) technique. The specific aim was to evaluate if the maxillary canines and first molars movements follow a curvilinear shape of movement rate. The secondary aim was to evaluate horizontal and vertical monthly movement rates of maxillary canines and first molars. The tertiary aim was to evaluate how similar cusp movement was to the apex of the same tooth. The null hypothesis was that maxillary canines and first molars movement rates follow a linear pattern over time.

## MATERIAL AND METHODS

This was a prospective study, conducted at the Department of Orthodontics at São Paulo State University (UNESP) - Araraquara School of Dentistry (São Paulo, Brazil), between February 2010 and December 2014.^4^ The study was design to evaluate possible differences in canine retraction rate, as well as changes in tipping, and the amount of anteroposterior anchorage loss during maxillary canine retraction. The Ethics Committee of the institution approved the study (ethical approval No. 65053917.2.0000.5416). All patients gave their informed consent, as required by the human subjects committee.

Twenty-five young adult patients (16 females and 9 males) were selected according to the following criteria: Brazilian males and females, age above 17 years (23.3 ± 5.1), bimaxillary protrusion, Class I malocclusion, mild crowding in the maxilla and/or in the mandible (≤ 4 mm), presence of all permanent teeth (except third molars), and no previous orthodontic treatment history. Subjects were not included if they presented poor oral hygiene, carious lesion, periodontal disease, systemic diseases or physical limitations.

The sample size was calculated using the PS: Power and Sample Size Calculation software, version 3.0.43 (Vanderbilt University, Nashville, TN). A sample size of 25 patients was calculated for a significance level of 5% and a sample power of 90%, based on estimated differences of 0.2 mm of space closing between periods and a standard deviation of 0.3 mm.^17^

All patients used stainless steel fixed appliances (Ovation 0.022-in slot straight-wire brackets, GAC, Bohemia, NY, USA) placed from the second molar to the second molar in the maxilla and mandible. All molars were banded. Leveling and alignment were conducted until 0.020-in stainless steel (SS) archwire could be passively inserted into the brackets. After that, the second molar to the second premolar of each side were tied together using a 0.010-in ligature wire, forming the anchorage segment, and then extractions of the first premolars were performed. The patients did not receive any additional anchorage devices.

**Figure 1:**
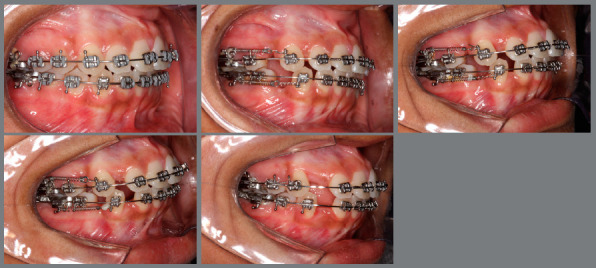
Canine retraction: initial (A), after one month (B), three months (C), 5 months (D) and 7 months (E).

Space closure mechanics began 7-14 days after the premolar extractions. All patients underwent orthodontic treatment with space closing by TSR (Fig 1). The canine retraction was undertaken on 0.020-in SS archwire with tight omega loops tied to the first molars on each side. NiTi closed-coil springs were activated with 100 gram-force (GAC, Bohemia, NY, USA) and connected from the hook of the first molar bands to the hook of the canine brackets with the help of ligature. The patients were evaluated every 28 days. During each appointment, all the springs were removed and checked by Correx Tension Gauge (Haag-Streit, Bern, Switzerland).

**Figure 2:**
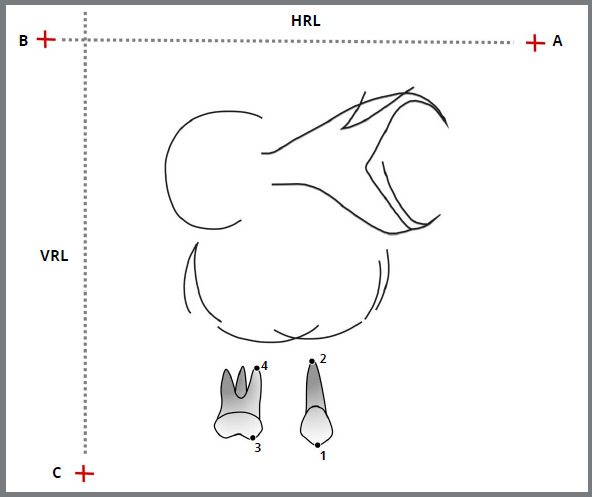
Cephalogram at 45º illustrating the landmarks and lines used in this analysis. Points: 1) canine cusp; 2) canine apex; 3) mesial cusp of first molar; 4) mesial apex of first molar; A) anterior point; B) posterior point; C) inferior point; Horizontal (HRL) and vertical (VRL) reference lines.

Oblique lateral cephalometric radiographs of both sides were taken immediately before starting the canine retraction (T0), after one month (T1), three (T3), five (T5), and seven (T7) months. Three fiducial reference points were marked in T0 cephalometric tracing (Fig 2). Two horizontal points were marked parallel to the functional occlusal plane, to determine the horizontal reference line (HRL): Point A, located in the anterior region of the tracing; and point B, located in the posterior region of the tracing. A third point, Point C, was marked above the orbit contour and posterior to the tracing, to determine a vertical reference line (VRL). Two landmarks were marked on both the molar and the canine in each traced cephalogram (Fig 2).

**Figure 3:**
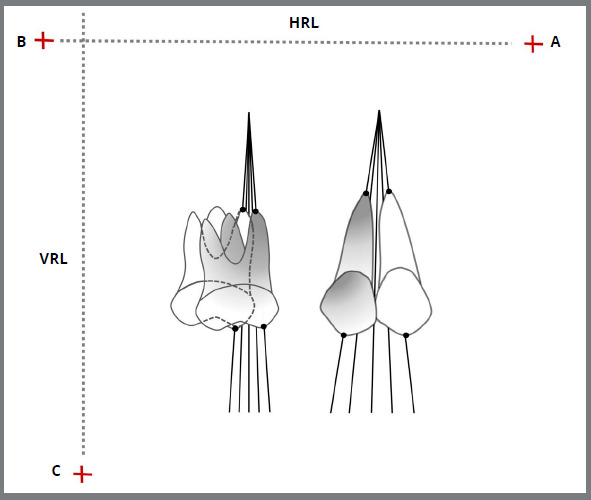
Cephalograms superimposition, with canine and molar movements during the follow-up time.

Partial maxillary superimpositions were performed on the best fit of the stable structures, using the inner cortical bone of the anterior part in the maxillary canine region at the contralateral side, the posterior contour of the infrazygomatic crest, orbital contour and nasal floor (Fig 2).^18^ The three fiducial reference points (A, B, and C) were transferred from T0 successively to T1, T3, T5, and T7 (Fig 2). DentoFacial Planner Plus^®^ (DFP; version 2.0; Toronto, ON, Canada) was used to digitize all radiographs. The horizontal displacement of the cusps and apexes was measured by the distance of their reference points to the VRL line (Fig 3). Similarly, vertical displacements were measured from these points to HRL (Fig 3). The amount of movements was measured from the origin, and the tooth movement rates were calculated for each time interval. A single-blinded investigator traced, superimposed, and digitized all the radiographs. To evaluate the systematic and random errors, 10 randomly chosen patients had all the radiographs (T0, T1, T3, T5, and T7) traced, superimposed, and digitized again after an interval of four weeks.

### STATISTICAL METHODOLOGY

Statistical calculations were performed using IBM SPSS software (version 25.0, SPSS, Armonk, NY, USA) and R Statistical Software (R Core Team, version 2018, Vienna, Austria). A sample size of 25 patients was calculated for a significance level of 5% and a sample power of 90%, based on estimated differences of 0.2 mm of space closing between periods and a standard deviation of 0.3 mm. To evaluate the systematic and random errors, 10 randomly chosen patients had all the radiographs digitized again after an interval of four weeks. The intra-observer random error was estimated using intraclass correlation coefficient (ICC) and method errors [√(∑d^2^/2n)],^19^ and systematic differences were assessed using a paired *t*-test.

After random and systematic errors, the next step of the statistical analysis was to determine the shape of each subjects’ average tooth movement rate curve that best fitted on the subjects’ longitudinal data points. Multilevel procedures were used to derive polynomials for each of the measurements. The shape of the curve was estimated by polynomial models. The basic models consist of fixed (the average growth curve for the sample) and random parts (the partitions variation between subjects at the higher level and variation between time of the same subject at the lower level). The models allow a unique growth curve to be derived for each subject, based on its deviation from the average curve. Curve fitting procedures were performed to minimize cephalometric errors.

After the shape of the curves had been determined, outliers and the data were assessed by the boxplot and Shapiro-Wilk test (p > 0.05), respectively. No outliers were found, and skewness and kurtosis were judged to be normally distributed. Means and standard deviations were used to describe central tendencies and dispersion. The horizontal and vertical movements were analyzed. Confidence Intervals (CI) of 95% were also calculated. A Student’s *t*-test was used to compare the variables between the right and left sides. A level of significance of 5% was adopted (α = 0.05). A one-way repeated measures ANOVA was conducted to determine whether there were statistically significant differences over the course of the 7-month treatment, in other words, if there was difference between evaluated periods of time. The assumption of sphericity was violated for horizontal movement rates, as assessed by Mauchly’s test of sphericity; therefore, a Greenhouse-Geisser correction was applied.

## RESULTS

Intra-observer systematic errors were similar. Of the 16 differences, only one was statistically significant (p = 0.019): the first replicate of 16_Cusp_V was larger than the second replicate (0.05 mm). Method errors ranged from 0.022 mm (23_Cusp_h) to 0.025 mm (26_Cusp_h). Interclass correlation (ICC), ranging from 0.925 to 0.999, showed excellent reproducibility.

Multilevel models indicated a more complex tooth movement rate for molars (Table 2) than canines (Table 3). Only three out of 16 measurements followed quadratic or second-order polynomials (curvilinear or change in rate), indicating that the horizontal tooth movement rate for 16_Cusp, 16_Apex, and 26_Apex decelerated over time. 

**Table 2:** Multilevel models describing the horizontal and vertical first molar movement changes between the beginning of space closure and the seventh month.

Horizontal									
		Constant		Linear			Quadratic		
Tooth	Units	Estimate	SE	Estimate	SE	Prob.	Estimate	SE	Prob.
16_Cusp	mm	0.29095	0.13040	*0.47420*	*0.06993*	*<0.001*	*-0.02175*	*0.00853*	*0.010*
26_Cusp	mm	0.57645	0.11995	*0.34420*	*0.06155*	*<0.001*	-0.00525	0.00750	0.484
16_Apex	mm	0.20880	0.11767	*0.43480*	*0.05672*	*<0.001*	*-0.02200*	*0.00692*	*0.001*
26_Apex	mm	0.23150	0.11801	*0.39600*	*0.06184*	*<0.001*	*-0.01750*	*0.00754*	*0.020*
Vertical									
16_Cusp	mm	0.02205	0.07987	-0.04820	0.03900	0.217	0.00475	0.00476	0.318
26_Cusp	mm	-0.04140	0.08898	-0.00040	0.04402	0.993	-0.00100	0.00537	0.852
16_Apex	mm	-0.05650	0.07729	-0.02400	0.03734	0.520	0.00050	0.00456	0.913
26_Apex	mm	0.00420	0.09092	-0.02280	0.04195	0.587	0.00100	0.00512	0.845
16_Cusp at 6 months (horizontal) = 0.29095 + [0.47420*(6)] - [0.02175*(6)^2^] = 2.3531 mm									

Bold and italic font indicates statistically significant (p <0.05).

**Table 3:** Polynomial models describing the horizontal and vertical canine movement changes between the beginning of space closure and the seventh month.

Horizontal									
		Constant		Linear			Quadratic		
Tooth	Units	Estimate	SE	Estimate	SE	Prob.	Estimate	SE	Prob.
13_Cusp	mm	0.57610	0.20240	*0.78560*	*0.11012*	*< 0.001*	-0.02450	0.01343	0.068
23_Cusp	mm	0.68900	0.22191	*0.60200*	*0.11831*	*< 0.001*	-0.00300	0.01443	0.835
13_Apex	mm	0.45285	0.18231	*0.30460*	*0.09517*	*0.001*	0.00475	0.01161	0.682
23_Apex	mm	0.65345	0.16459	*0.21420*	*0.08704*	*0.014*	0.01175	0.01062	0.268
Vertical									
13_Cusp	mm	-0.26830	0.10570	0.07120	0.05497	0.195	-0.00450	0.00670	0.502
23_Cusp	mm	-0.13230	0.10233	-0.04680	0.05357	0.382	0.00350	0.00653	0.592
13_Apex	mm	-0.19560	0.08234	0.01240	0.04089	0.761	-0.00200	0.00499	0.688
23_Apex	mm	-0.15575	0.09769	-0.04500	0.04358	0.302	0.00175	0.00531	0.742
13_Cusp at 6 months (horizontal) = 0.57610 + (0.78560*6) = 5.2897 mm									

Bold and Italic font indicates statistically significant (p < 0.05).

A Student’s *t*-test showed that there was a significant difference in 3 out of 32 comparisons between right and left sides. All three differences were related to the vertical movement of canine cusps at the third month (p=0.032), fifth month (p=0.016), and seventh month (p=0.024). In this way, the results for the canine and molar movements of the right and left sides were grouped by the average.

**Figure 4:**
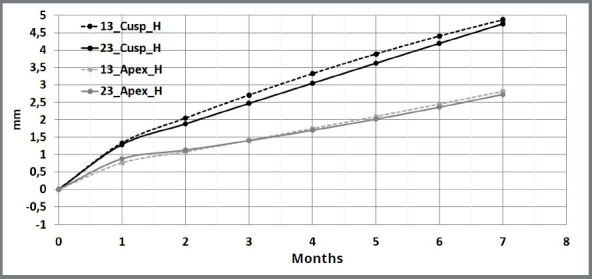
Descriptive time-movement curves of canine cusps and apexes during horizontal movement.

Canine cusps showed significantly more total horizontal movement than canine apexes over a 7-month period (Fig 4). In addition, the total difference between horizontal canine cusps movement and horizontal canine apexes movement increased over time. Canine cusps presented monthly movement rate larger than canine apexes in all four evaluated period (Table 4). 

**Table 4:** Descriptive statistics and statistical comparisons of the horizontal movement rates per month.

		Cusp		Apex		Cusp - Apex	
Tooth	Month	Mean	SD	Mean	SD	Mean diff.	Prob.
Canine	T1	1.32	0.52	0.81	0.43	0.51	*<0.001*
	T3	0.62	0.24	0.31	0.18	0.31	*<0.001*
	T5	0.62	0.30	0.31	0.17	0.31	*<0.001*
	T7	0.51	0.20	0.37	0.23	0.14	*0.002*
Molar	T1	0.82	0.26	0.61	0.21	0.21	*<0.001*
	T3	0.37	0.16	0.35	0.13	0.02	0.494
	T5	0.28	0.12	0.24	0.12	0.04	0.099
	T7	0.26	0.13	0.19	0.10	0.07	*0.003*

Bold and italic font indicates statistically significant (p < 0.05). Mean difference (Mean diff.) was calculated as Cusp minus Apex.

Horizontal canine cusps showed a larger movement rate at the beginning of canine retraction, followed by significantly smaller and constant movement rate after the first month (Table 5). The same horizontal pattern was observed for the canine apexes. 

**Table 5:** Statistical comparison of the horizontal tooth movement rate among each of the observed period.

Tooth	Month		Cusp		Apex	
			Mean diff.	Prob.	Mean diff.	Prob.
Canine	T1	T3	0.70	*<0.001*	0.51	*<0.001*
		T5	0.70	*<0.001*	0.51	*<0.001*
		T7	0.81	*<0.001*	0.44	*<0.001*
	T3	T5	0.00	0.999	0.00	0.999
		T7	0.11	0.999	-0.07	0.999
	T5	T7	0.11	0.884	-0.07	0.999
Molar	T1	T3	0.46	*<0.001*	0.26	*<0.001*
		T5	0.54	*<0.001*	0.37	*<0.001*
		T7	0.57	*<0.001*	0.42	*<0.001*
	T3	T5	0.08	0.499	0.11	*0.017*
		T7	0.11	0.153	0.16	*0.001*
	T5	T7	0.03	0.999	0.05	0.766

Bold+italic font means significant difference (p<0.05). Mean difference (Mean diff.) was calculated using the observational month on the left month column minus the observational month on the right month column. Example: canine cusp in the month 1 - canine cusp in the month 3.

**Figure 5:**
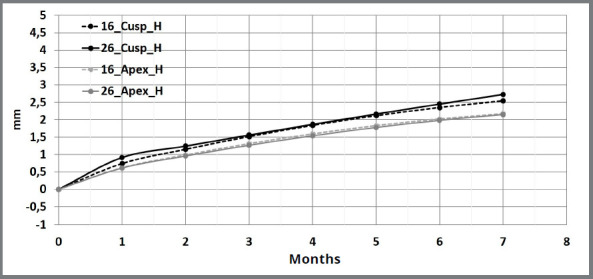
Descriptive time-movement curves of molar cusps and apexes during horizontal movement.

Molar cusps also presented more horizontal movement (2.64 mm) than molar apexes (2.17 mm) over the seven-month evaluation (Fig 5). The differences between horizontal molar cusps movement and horizontal molar apexes movement increased from 0.21 mm in the first month to 0.47 mm in the seventh month. Molar cusps showed a larger movement rate than molar apexes only in the first (0.82 mm/month vs. 0.61 mm/month) and seventh (0.26 mm/month vs. 0.19 mm/month) months of evaluation (Table 4). 

In general, molars showed a similar horizontal movement rate pattern than canines, presenting cusps with larger movement rate at the beginning of the canine retraction followed by significantly smaller and constant movement rate after the first month (Table 5). At the beginning of the canine retraction, molar cusps presented an initial movement rate of 0.82 mm/month, decreasing significantly to 0.37 mm/month at the third month, with a constant movement rate of 0.28 mm/month and 0.26 mm/month at the fifth and seventh months, respectively. 

Canines’ cusps showed more total horizontal movement than molars cusps (4.80 mm vs. 2.64 mm) and larger monthly movement rate for all the evaluated periods. 

## DISCUSSION

**Figure 6:**
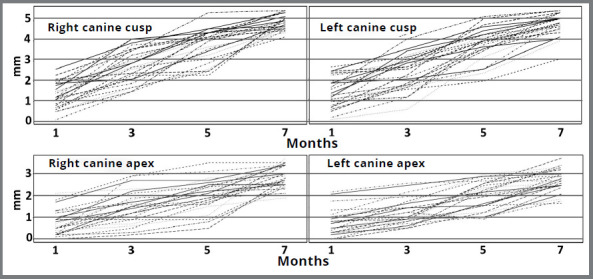
Estimate canine horizontal movement for each of the individuals by multilevel models.

The present study was the first to evaluate horizontal and vertical tooth movement using multilevel polynomials. Perhaps the most interesting outcome of the present study relates to the fact that, in general, molars followed a quadratic curve (curvilinear decreasing, or deceleration, over time) and canines did not (linear decreasing) regarding horizontal movement. It is also important to notice large subjects’ variation during canine retraction (Fig 6), which could mask the possibility to visualize the quadratic pattern. Animal studies showed an immediate slight tooth movement, followed by a lag phase, and then an accelerated rate,^18,20^ but human studies are inconsistent.^17^ ^,^ ^21^ Regardless of mechanics, the rate of tooth movement has also shown a large variability among individuals, ranging from 0.2 mm/month^22^ to over 2.5 mm/month.^23^ This wide range could be due to the study design, such as space closure based on friction or frictionless mechanics or lack of movement control (i.e., uncontrolled tipping).

Canine cusps presented a higher movement rate at the beginning of the retraction. At the third and fifth months after canine retraction beginning, the canine cusps showed approximately 53% of the movement rate observed in the first month of canine retraction. The movement rate decreased even more at the seventh month of canine retraction, with canine cusps presenting only 39% of the initial amount of retraction. On the human studies, there were some inconsistencies regarding canine retraction rate decrease overtime under friction mechanics^9^ or increase after the first four weeks under frictionless mechanics.^17^ However, the former study^9^ did not provide statistical analyses, and the latter^17^ evaluated the subjects only for 8 weeks or two follow up appointments. An animal study showed a clear pattern for tooth movement, with an initial tooth movement lasting 3 or 4 days, followed by almost no tooth movement for approximately 7 days, and accelerated tooth movement, with later constant and less tooth movement rate.^20^ Most studies on human subjects could not provide sufficient information regarding the lag phase based on their tooth movement rates.^21^ ^,^ ^24^ Study designs and various biological and biomechanics factors could explain the variability and inconsistencies between the canine retraction movement rate in the literature, making comparisons problematic. 

Understanding how teeth move is the basis for providing a more efficient treatment, due to better mechanics choice. Some biological factors, such as the regional acceleratory phenomenon (RAP), can influence the larger initial movement tooth rate followed by a smaller tooth movement rate.^25^ This phenomenon could be generated due to the healing of alveolus right after tooth extraction. It has been shown that the tooth movement rate into recent extraction sites is higher than into healed extraction sites.^26^ In the present study, the space closure started just 7-14 days after the extraction of the premolars, which could contribute to a higher tooth movement rate at the beginning of the canine retraction.

Canine apexes showed larger movement rate at the beginning, which decreased, with no or slight changes overtime, during canine retraction. The literature is scarce and only one study evaluated the canine apex movement rate using lateral cephalograms. During frictionless canine retraction, it was shown that canine apexes movement rate was minimal, and the apexes were maintained in place over 8-week; smaller movement could be expected due to a higher controlled movement of the T loop regarding moment-force ratio delivered.^17^ Thus, it can be suggested that the moment-force ratio can modify the type of tooth movement, such as controlled tipping. Then, type of tooth movement has relevant clinical influence in the amount of the tooth movement achieved.

The molar cusps showed mesial displacement at the beginning of the canine retraction. Like canine movement, molar cusps and apexes also presented a higher movement rate at the beginning of the retraction. The third month after starting canine retraction, molar cusps and apexes showed approximately 55% of the movement rate from the first month, decreasing even more over time (as little as approximately 30% at the seventh month). Molar apexes and cusps presented similar horizontal movement rates, showing that they closely translate mesially during canine retraction. Only one study evaluated the molar rate movement during canine retraction, but using maxillary dental casts did not allow the evaluation of the molar apex movement.^8^ However, the finding cannot be directly compared because this study used transpalatal arches and the patients were still actively growing.^8^ It has been reported that monthly molar anchorage loss rate is approximately 0.46 mm/month (Table 1).^5^ ^-^ ^12^ Previous studies have shown that when the retraction was associated with anchoring devices, such as miniscrews, the molars showed minimal movement and there was a decreased in retraction time.^27^ ^,28^

This study used forces of 100 g applied with nickel-titanium coil springs on a 0.020-in SS archwire, because the literature has shown efficient tooth movement with these parameters. Comparisons are problematic due to the great study design variations. Some studies used different sources of force, such as elastomeric chain^5^ ^,^ ^7^ ^,^ ^8^ or NiTi closed-coil springs.^5^ ^-^ ^12^ Due to its superelasticity property resulting in a more constant force, when compared to elastomeric chains, nickel-titanium coil springs were used.^29^ Regarding the amount of force, most of the studies have reported effective tooth movement with light forces (100-200g).^5^ ^-^ ^12^ Archwire cross-section also has influence on tooth movement rate. Flexible archwires such as 0.016-in SS allow more canine movement than 0.020-in SS over 10-week period, but canines present three times more tipping (5.30±2.37° and 1.70±1.35°, respectively).^30^ ^,^ ^31^ Since less tipping can occur on the 0.020-in SS archwire and light continuous forces of 100 g provides tooth movement rate similar to the one reported in similar studies, there appears to be an advantage in retracting canines using this study design.

It is important for the orthodontist to have in mind the type of mechanics to be applied during canine retraction. Due to cusps and apexes horizontal movement amounts, this study showed that canines presented tipping instead of bodily movement. Differences between the type of tooth movement are clinically relevant,^3^ which could explain higher canine movement rate at the beginning. The present study also suggests that individual canine retraction produced anchorage loss when no anchorage was planned or applied. Maxillary canine cusps were retracted 4.8mm, while the maxillary molar cusps mesialized 2.64mm (35.4% of anchorage loss). Interesting was the fact that approximately 60% of the molar mesial movement or anchorage loss was obtained at early stages of canine retraction. A randomized clinical trial showed that *en-masse* and two-step retractions presented no difference in anchorage loss, with slightly less anchorage loss (15%) for the *en-masse* group.^32^

## CONCLUSIONS

This study failed to reject the null hypothesis regarding horizontal canine movement rate, but not for the horizontal movement rate. When the cusps and apexes movement of the maxillary canines and first molars were analyzed during the first step of extraction space closure associated with the two-step retraction technique, the following conclusions could be drawn:

» Canine did not accelerate or decelerate overtime horizontally.
» The cusps and apexes of the canines and molars showed a larger horizontal movement rate at the beginning of canine retraction, followed by significantly smaller and constant movement rate after the first month.
» The canine and molar cusps showed more horizontal movement than canine and molar apexes over a 7-month period.
» Canine cusps and apexes showed more horizontal movement than molar cusps and apexes.
» Canine and molar cusps and apexes did not show clinically significant vertical movements.
» Canine retraction produced anchorage loss.
